# Supplementary material for: SEVtras delineates small extracellular vesicles at droplet resolution from single-cell transcriptomes
Source: Nat Methods. 2023 Dec 4;21(2):259–66. doi: 10.1038/s41592-023-02117-1 (PMC10864178; doi:10.1038/s41592-023-02117-1)
Supplement: Supplementary file 1 — Supplementary Tables 1–4. [file 41592_2023_2117_MOESM1_ESM.pdf]

nature methods

Article https://doi.org/10.1038/s41592-023-02117-1

SEVtras delineates small extracellular vesicles at droplet resolution from single-cell transcriptomes

In the format provided by the authors and unedited

Supplementary Tables

Supplementary Table S1: SEV-gene set

**sEV genes**	SNORD113-7	RLF	SLC33A1	RBM24
CUL4A	PTP4A2	GNB4	SMC1A	SSTR1
IKZF2	SNX17	IRF3	TRU-TCA1-1	ACTB
SNORD117	PES1	NCBP1	RN7SK	OR51B6
SF3A1	CCL20	KBTBD3	DCTN2	PPP6R1
MAP3K13	ADI1	TRIOBP	SULT1A3	MUC16
RPS19	RPPH1	EIF4A1P10	RNF123	TSFM
LRRC58	C6orf62	PARP1	MIMT1	STRAP
CSK	EIF1	RNA5S17	NCKIPSD	AFAP1-AS1
FAM21A	GPI	AXL	RNY4P31	ALB
FAT1	FTSJ2	NUFIP2	RAC1	COL5A2
ZBTB20	NOL12	CPLX1	USP46	CATSPERG
AKAP12	SYNM	RPL13AP5	HYAL1	RNF25
CD55	FGG	SNORD67	THOP1	FAM111B
ACOT7	RNA5S7	PRDX4	TLN1	HIST1H3B
COL8A1	TRD-GTC1-1	TRIM68	SNORD121A	LARP4B
CLTC	HIST1H2AG	RNU1-28P	RAP2A	UBE2B
WDFY1	TRX-CAT1-5	BRD4	RNY4	SNORA19
RPS12	GTPBP4	ARID1B	SNORD48	SEC14L1
ST3GAL4	ESAM	DDX19A	SNORA45A	SMC5
CWC27	RB1	PABPC4	TBC1D14	RNU1-4
SNORD89	TTLL12	RPL18AP3	STRN4	FOXN3
RNA5S3	SIAH2	KCTD1	ARNT	SNORD91B
EIF4G3	SNORA71D	BRD3	SNORD114-17	RBM44
KHSRP	ZNF672	DBI	RPL37	SNHG3
BRD8	THRB	CSDE1	SNRPD2	DIMT1
KIF14	RAP1GAP2	MEGF9	STAT6	CNDP2
CRIM1	TRK-TTT3-5	SDHA	NRIP1	NOTCH3
ARRB1	AZIN1	PLEKHA6	RPH3AL	RNU1-27P
MTHFD2L	NME1-NME2	SETD1A	HES7	PABPC1
C9orf78	LBR	TRG-GCC1-2	AHR	TRV-TAC1-1
TPD52L1	TRAF3	HSBP1	MMP1	SNORA78
AIFM2	SCARNA6	PLEKHB1	BAG5	GCNT2
MCM8	TFF1	EPT1	NBAS	CCNL2
RECK	WSB1	CD2AP	HHIPL1	DDX11
CRLS1	POTEF	MARS	SNORD7	HIST1H1B
ETF1	TAF6L	AKR1B10	RPL28	RNA5S5
SLC25A32	KCNJ12	TNKS2	LCAT	HIVEP3
NFYA	ZDHHC14	KDELR2	CRTAP	QPRT
LIFR	TMEM2	FAM171A1	ZRANB1	MICAL3
ZFP36L2	ERI2	RPL15	TOPBP1	RNY4P17

PRR18	SMCR8	KIF13A	RYR3	PTK7
TIMP3	SNORA74A	NADK	CSNK1D	TTC37
ZMYND11	MLF2	SLC7A11	GDPD5	CCDC134
FNDC4	ATXN7L3B	SGOL2	SLC4A2	ITPR2
TRRAP	MYH9	AKT1	BRI3	RTEL1-
TRL-TAA1-1	H2AFX	TXNRD1	CCNY	TNFRSF6B
VBP1	PEBP1	RNA5S1	FAM46C	WWTR1
MKNK2	ISG15	RPL39	KDM2B	ZNF358
NDUFB7	RPLP2	SNORD114-21	TOR1B	PDLIM5
RNF152	FAM19A5	CENPB	SSR3	USP31
S100A16	THBS1	SH3TC1	SNORD82	GRB2
NMT2	ZNF451	C16orf72	RNU11	ATP2A2
VAMP3	ANKZF1	ALKBH7	INF2	MAEA
MCM7	EWSR1	ATP5A1	LPCAT1	B4GALT1
OAF	PDIA4	SUMO1	OTUD1	ZNF473
TRI-TAT2-3	MCM4	EIF3H	AKT1S1	STARD7
TPR	ZMYND10	CRYGS	KIAA1468	STX4
PSMB3	ATP5D	RERE	VTRNA1-1	ABCA3
MGP	SREBF2	ARHGEF11	INPP1	MALAT1
MCM6	CCDC57	IMPDH2	MIIP	TRA2B
HINT1	RN7SL2	SRSF6	USP34	STK4
GAN	HUWE1	MAP7D3	DDX24	LASP1
IRF2	SNORD11	TRD-GTC2-11	EIF4B	UBE2Q2
GLUL	DDX3X	ZCCHC14	SEC22B	POLR2A
ZNF629	ZNF556	LAMC1	LCN2	MAGT1
TMEM123	TRIO	E2F1	NME1	GJA1
PRNT	UHRF1BP1	LRPPRC	BCL2L13	CERK
LAMTOR1	GNAI3	TUBA1B	LMO7	SNORD114-9
SOX5	IGF2R	CDCA3	SNORD60	RAN
SUPT16H	IRF2BPL	DOK4	POM121L10P	RNY1
AK3	FBN1	CS	MRPL32	URGCP-
HIST1H4L	LNPEP	MAPKAP1	PITPNM1	MRPS24
DPP10	AFAP1	KCTD15	DYNC1LI2	SDHAF2
TRD-GTC2-7	PTPRJ	AMFR	NEDD8-MDP1	ZNF317
CHST2	FAM193B	PPP4R1	RPAP3	SCARNA12
TRD-GTC2-2	MAP1B	SNORD66	DNAJA2	UBE2D3
PTPRA	SNORD101	RNA5S15	SLC11A2	DMRTA1
BPGM	PRPF40A	MYO5C	NKD1	FKBP11
CAMK1D	MRPL51	SNORD114-3	N4BP2L2	MLH1
MON1A	PPM1G	AMMECR1L	TRC-GCA1-1	MSN
MMGT1	AP2A1	RBMS2	SNORD37	SNUPN
GOLGA8A	CIC	NDRG2	SKI	SNAPC3
DHRS2	PDS5A	COX19	SRSF8	SLC35E2
CIAO1	TUBB4B	U2AF2	GLCCI1	RBM15

TRE-TTC2-1	HIST1H3H	BICD1	PARP4	ATP6V1B2
DUS1L	SLC25A3	DIRAS1	FASN	NPEPPS
CAPZB	HECTD1	FHDC1	DPYSL2	THOC5
SNORD8	EIF5A	HIST2H2BE	RASSF5	MTA2
EFHD2	ETFA	POP4	EIF2S2	ATG4B
PURB	HIST1H2BB	EP300	COASY	LGR4
CCNI	BACH1	CHKA	RPL35	EIF4EBP2
RPL22L1	TOR1AIP2	UBE2V1	DHX15	S100A6
IFIT2	FBXO31	BEAN1	MRPL45	AAMP
MYH7	TRR-ACG1-2	FBXO2	SNORD22	HNRNPA2B1
RNU5E-1	GBE1	ITPR3	RNA5S4	PDCD6
HRAS	PPP1R8	RNU6ATAC	RORC	ELOVL1
NACA	BAIAP2L1	GRB10	AHNAK	CLDN4
RNF167	ANTXR1	FBL	LYRM4	TUBB
PSMA7	TOP1	FAM96B	ZBTB7A	KCNJ2
AFF4	TK1	CD63	USP7	PPA1
UBXN7	CDYL	DLC1	TMEM181	FUBP3
FOXO3B	ROCK1P1	SCARNA11	ETFB	MYL12B
AGR2	SNORD114-28	MRPL28	MIB1	DCAF13
AKAP13	NOTCH2	SNHG7	CRK	DYNLL2
ACSF3	HIST1H2AL	TRV-CAC3-1	CDC42EP3	FARP1
RAI1	KLF9	TMEM245	UBE2E3	ZBED4
FYCO1	INS-IGF2	DPH2	SNORD92	RPL29
NAB2	SERPINB9	PNMA1	GALNT2	RPS8
GGCT	ERRFI1	ASPM	EIF3D	PTBP1
RAB3B	SLC25A13	TTC28	ARPC4-TTLL3	GPR107
C9orf116	PTPN11	B2M	TRH-GTG1-3	SQSTM1
CORO1C	DGKH	SETD1B	TMED7	MTCH1
LCOR	HIST1H3I	TRIB2	ZCWPW1	HIST1H4E
ITGA6	ANXA2P2	SOCS3	HDAC8	SMARCA5
EIF3CL	FAM45B	HAUS5	PAPD4	AXIN2
CYB5R2	NCOA2	HIST1H4C	AFAP1L1	FBXO38
ASB1	CEP68	SYK	SHMT2	WSB2
RPLP0	PIP4K2B	TRPV2	HIST1H2BI	RAB27B
SH3BP5	TRG-GCC1-1	SNORA74B	WDR1	ZNF155
KCNMA1	SNORA79	CHST15	STOML2	NF2
GRIPAP1	PARS2	NDUFS5	CYFIP1	THRAP3
RAB13	RHBDD1	KEAP1	FOXK1	NDUFA1
RNU1-2	NANOS1	CT45A3	DENND1A	SNORD26
ADM	SPCS3	MYL12A	PHB	CCNB1
HLF	SNORD99	YARS	OSBPL9	ZNF217
DENR	DCAF6	KDELR1	TNFAIP2	RNU1-3
SUGT1P3	CENPV	CPSF1	OTUD5	GULP1
LRRC59	HS3ST3B1	DNAH3	THAP4	COL21A1

SNORD127	CARM1	SNORD53	RPL3	DDX6
IL34	RTN3	PGRMC2	TGM2	ECT2
TPI1	SNORD114-14	CACNA1B	IRS1	DLST
MXI1	TUBA1A	GOLPH3	DDX21	SNORD78
IQSEC1	SPINT1	NRAS	NOP56	MRPL18
PTPN1	HBA1	CAV2	C22orf46	NCEH1
ENO3	CDCA4	NCAPD3	DDIT3	FRAS1
SAT2	KLB	HIST1H4B	MYPN	FCGR2A
C20orf27	HNRNPUL1	RAP1GAP	PDPR	DST
TSPYL1	SLC9A3R1	DDOST	VIM	UACA
HMGA1	CDC25B	BRCA1	APBA2	KCTD20
TACC2	PRPF6	YY1AP1	CAMK2G	GSK3B
KCMF1	MKL1	RPS4Y1	RANBP2	PDXP
BCAR1	ZNF326	CLIC4	BTBD10	SURF4
RTEL1	RAP2B	CCT3	TRD-GTC2-9	CBX5
LRP1	KRT80	NDST1	G3BP1	PSMD9
TRD-GTC2-6	ANP32B	PLA2G2A	PTPRS	EEF1G
APOA2	SH3BP4	TMED5	HIST1H1D	HIST1H3F
RNY4P27	TESC	SNORD12B	LYPD3	SLITRK1
TOR3A	KIF3B	FZD5	PRKCB	NEK6
SETD5	SNORD59A	FBXW4	LZTS2	ILF3
MED13	SLC50A1	IPCEF1	SMYD5	MLLT4
NHP2L1	PARG	GDF15	SNHG5	TMEM43
KIRREL	GAB2	CUL3	ARL8A	PCSK7
TRV-TAC2-1	ANKRD11	ASCC1	NSF	SNORD17
ELAVL1	USP22	SCARNA2	ST5	HNRNPH3
KLHL2	MGST1	HIPK3	RRP1	ARL4C
HPS4	DCLK2	POGK	TRV-TAC1-2	PSMB2
EIF4A1	VAPA	SNORD19B	LDHA	MDGA2
HMOX1	BRD1	TMEM107	P2RX5-	SLC2A6
PTPN9	RFC3	PRKDC	TAX1BP3	CAMLG
ITSN2	VAT1	TAF6	RTN4	CHID1
PRDX3	DBN1	ADRM1	HPCAL1	CTBP1
TRX-CAT1-2	HDGF	HIST3H2A	ATF7IP	VAMP8
DENND4A	MAP1LC3B	PPP2R1A	USF2	DTYMK
KIF1C	TPM2	TRIM25	RAD54L	HIST2H4B
UBE2K	TRABD	LRFN5	MAN1B1	TANK
TFRC	TBC1D30	SNORA25	C2orf49	SERTAD2
CPSF6	HGS	SHANK2	NFIX	KCNC3
WIPF1	SNORD1B	RAB11FIP1	PDHB	TCP1
STX8	ASRGL1	TRX-CAT1-1	TRD-GTC2-3	CLIP2
BTRC	CCT2	PSMD13	IQCE	CEP55
RPL7A	SNTB2	HIST1H2AE	TRK-TTT3-2	VMP1
POLR2L	SYNPO	SLC9A1	S100A13	ITM2B

ELOVL7	SNORD14E	ZNF652	TMSB4X	RNF114
LYST	BCL2L2	ENO1	PLD2	CDKN2B
NFE2L3	SNORA7B	ELF4	HSPA5	ZNF674
DGKD	APOB	TRD-GTC2-8	HOXB6	MARCKS
CXorf38	SERP1	UBTF	SCARB2	NUP98
PCDH19	DISP1	OSBPL1A	SGSH	RPL31
TSNAX-DISC1	SNORD69	NUP50	TRIB1	ABL1
EEPD1	TFAP2C	DDX60	SNORD93	CCDC88C
RNA45S5	HSPD1	RPL23P8	IGF2BP1	TACC1
UGCG	LAMB1	LHFPL2	MBD5	STXBP3
RASSF3	WASL	RPL4P4	RHOB	COG3
LAMP2	HIST2H4A	ATP6V0C	ALDOA	CHP1
KIF11	TRG-GCC1-4	RPL18	MAN2A1	RBM25
FANCC	SCD	RABL2A	GORASP2	BTD
ITM2C	EML2	ADA	SNORD74	DCUN1D2
DNAJB1	HIST1H2BN	ZNF335	SNAPC1	H2AFY
ADIPOR2	RAPH1	DEAF1	PGM5P2	ATP5G3
MYO16	TRK-TTT3-4	ALDH1A1	EIF3L	GABRB3
CLDN7	POC1B	ATP5B	CIRBP	TPCN1
NAB1	PHTF1	GPATCH4	FAM134C	CAND2
SNORD43	RNF157	PIGY	HIST1H1E	SNORD72
AP3S2	SEMA6B	VWF	HEATR1	PANK4
PHLDA1	ATXN7L1	RPS20	CTNNBIP1	SNORD112
STT3B	CHD8	TRH-GTG1-7	PTPRB	TAF4
NFE2L2	BOD1L1	HIST1H1C	STAT5B	SLC35E1
RTKN	YKT6	SNORD38A	SNORD14C	GXYLT1
SLC30A1	WWC3	UBAC2	CTTN	SNORD85
HEXIM1	PC	GOT2	PPFIA3	SNORD114-20
PRMT1	WDR37	FBRS	TRH-GTG1-1	SNORA75
TRNAL-CAA	VDAC1	CALM3	TRIM41	PGK1
SCARNA10	RPL27	FAM199X	EIF4A2	AKAP11
RNU1-1	ELF1	SARS	FOS	AP4S1
SPATS2L	RPL32	HNRNPD	NEB	RBBP6
HIST1H4A	ZNF37A	C9orf3	EXTL2	NMRAL1
H1F0	TRAM2	FSTL1	AGPAT6	TRR-ACG1-1
CALM1	PPP4C	DOCK1	CBS	SNORD105B
TPT1	RHOT2	FLNB	RPS15	ANKRD1
VTRNA1-3	SLC23A2	NT5DC2	ELK4	SLC38A2
PFN1	SFPQ	GTF2F1	FARS2	POGZ
IGFBP5	CSNK1A1	FAM120A	DCBLD2	RNA5S9
SNORD114-15	IPO9	GTPBP2	MAFK	ZNF395
RPL8	HEG1	EPS8L2	ACTG1	TCEB3
TRIM35	RNA5S2	ZC3HAV1	RAB22A	PTCD1
DUSP10	TRR-ACG1-3	MRPL20	UBE2I	FHL1

ARHGDIA	PANK3	HIST1H2AI	TRH-GTG1-4	SNORD114-23
WDR12	VAPB	TRX-CAT1-3	TXNDC11	ALDH18A1
NUP188	MCFD2	TRL-CAA1-2	TRAK2	PDZD8
PNKP	RNA5S14	TUG1	FAT2	NUCKS1
BANF1	TBX3	DUSP6	WNK1	EGFEM1P
PAK2	GPX2	PLEKHM2	HIST1H2AH	SNORA26
PTGFRN	ZNF768	REST	STAB2	NRXN3
ENTPD4	NANS	GUSBP4	SCNN1B	GLA
RNY5	CDC5L	COX5B	HIP1	SEPT9
PACSIN3	EFNB2	TRL-TAG2-1	TUBGCP3	PSMB4
RNU4-2	SOD1	RP1	FOXP2	RRP36
CAPN7	SLC30A7	MYBL2	TRNS1	BAIAP3
ALDH3A1	EIF4ENIF1	SPG21	RPTOR	OSMR
RBM14	TOP2A	SMG1	EIF2AK2	KRT19
CD164	ABR	RLIM	BSPRY	SLC25A30
DUSP14	SNORD113-9	SLX1B-	RPUSD4	HBS1L
ATP1A1	PRKCH	SULT1A4	KCNIP4	PLA2G4A
OAZ1	UBA1	RPH3A	CIT	XAB2
RFX3	WBSCR28	RMRP	RPS2	MXD4
OIP5-AS1	RARS	SLC25A5	KBTBD2	RPL13
HNRNPC	PHF14	IFT122	ANKRD17	SON
PARP3	RNF213	MVP	NAPA	PTPN12
SNORA65	ASNA1	GNA12	TRH-GTG1-6	ZBED6
GPX4	HSPA8	MYNN	EEF1A2	GSR
USP3	RBM14-RBM4	CSNK2A2	TWF2	WTAP
NFKB1	SNORA71B	SNORD83A	SH3BGRL3	TRG-GCC1-3
SNORA77	SNORD114-1	STAT1	PRX	ART1
ZNF480	ATP6V0A1	HIPK1	ZBTB34	HIST1H4D
SNORD70	FAM168B	ZBTB2	GUK1	AHSG
ZBTB38	DOCK4	ARL6IP1	CCT5	JUN
SNORD113-3	ZCCHC2	CDCP1	C18orf25	SDK1
SNORD12	SHROOM4	IFITM3	NDUFB1	FMNL1
H2AFV	SPEN	YBX1	PYGB	CCDC6
RNA5S6	PGCP1	MCM9	ZUFSP	FZD4
METRN	CXXC4	SLC25A39	PFAS	SIRT5
ATP11C	SLC8A1	GTF2I	FGB	CELSR1
PEG10	SACM1L	SNORD12C	SEL1L	SSR4
VTA1	SNORD114-12	VPS37D	CDC42BPG	SNX10
ELF3	HSD3B2	IRS2	TNRC6A	NUBP1
RPL35A	PRMT5	TRG-GCC1-5	MYL9	UBR4
ADAM11	DDX5	TRE-TTC1-2	HIST1H3C	ARPC5L
PCBP1	PSMD11	RNA5S11	TBL1X	KLC2
SOS2	CDK6	IMPA2	SNAR-A9	DOCK9
EXOC5	CDC14B	LSM5	TRAF4	APP

SAMHD1	FOXP1	SNORD105	HERC2	DLG1
TNFRSF10D	SHFM1	SNORD64	RNU4ATAC	PFDN4
TXNL1	SLC1A5	CHD1	ANKRD40	SPTLC2
IMPDH1	MAP2	SNAR-A11	ARHGAP33	ASGR1
AES	ZMIZ1	CNOT4	TXN	SMPD4
MMAB	SNORD88A	PIAS1	EIF5AL1	RHOG
CACNA2D4	SCCPDH	BLOC1S2	WDR74	AKR1B1
H19	HSPA4	CHD2	CROCC	DOT1L
RBMX	BAGE	SNORA73B	TRX-CAT1-4	PFKFB3
TSC22D2	TM9SF3	AARS	NT5E	ARHGAP26
ARHGAP17	NAP1L4	KCTD10	AP1G2	SASH1
HIST1H3D	PITRM1	SNORA31	FTLP3	NQO1
TPM4	TTI2	CACYBP	PTAR1	CENPF
CDKN1B	TNKS	DMD	RPL30	LARP1
IQGAP1	IMPAD1	TTC3	CEP78	OTUD7B
ZNF850	RPSAP58	RND3	POLR2I	KLF2
PI4KA	HIPK2	AGPAT1	PAPD5	MTOR
SASH3	UNC119B	RAPGEF1	GTF3A	ROCK1
JMJD6	PRDX1	UBE2F-SCLY	CD44	OPN4
SOD2	JUND	YPEL2	BAP1	SNORD100
NHP2	CCDC69	PPP2CA	CTGF	TRE-TTC2-2
SNORA81	TKT	C1GALT1	FBXO18	COG1
RNU6-59P	CPSF2	CTSB	TOMM22	FAM208B
RHOA	CYBA	DDX54	CYR61	SNORD91A
GNB2	RPS11	ASPH	GET4	CD59
RPL23A	EPHB4	BTN2A1	ACIN1	JAK1
NAA35	SNORD51	NACC1	IGF2	HSD17B10
RPS5	MIDN	CPSF3L	POLR2H	IPO7
C5orf24	PPP1R14B	TRH-GTG1-8	SNORD11B	NFATC3
HNRNPAB	SNORD95	UBE2R2	PRDM13	PHF11
SNORD63	HIST1H2BO	UGGT1	TDG	KLK6
MEPCE	PRKACA	TRK-TTT3-3	UBASH3B	FAM83H
SRM	RNA5S10	TAF15	CLK2	ABCD3
P4HB	ECSIT	NDUFA4	COBLL1	DCAF4L2
PIK3CD	TAX1BP3	RIC8A	DPP9	EDC4
SERAC1	LMAN1	DDB1	SP5	MPZL3
TOR1AIP1	PAK6	ARAP1	GIT1	FGF2
MGAT4B	CPD	TIMP2	GMPR2	WDR11
WASF2	ANO4	RABGGTA	CDH2	ZNF516
NUP62	ERBB2	WDR26	IFT27	ANXA6
COPS2	ANXA1	TRH-GTG1-5	EXT1	IMMP2L
EEF2	PCYOX1L	SUV39H2	RAB5C	PTX3
ZC3H13	PCBP4	TMCO4	EHD1	SLC35D2
SNORD119	ZNF770	DYNLL1	CCAR1	HSPA1B

IL17D	RNF113A	APOA1	FEM1B	UBC
MET	RPL5	NFYC	KIAA0232	KIF5B
PPP1CB	EYA4	SORBS1	DYNC1H1	CHST8
LYPD6B	C1orf43	DKK1	CPNE1	RNU5A-1
TAB3	AGPAT5	SMAD2	ARHGAP35	PKD1P1
XRCC6	PKP4	ACTR2	ARHGEF3	CCND1
S100A4	SNORD114-26	GRB7	SNORD15A	RDH10
FAM83D	RPL37A	KRT8	CAMSAP1	ZNF318
TRK-TTT3-1	PEG3	EEF1A1	WBP2	URB1
KPNA3	UBE4B	PTRF	STAU1	PHLDB1
ERGIC1	EML4	SNORD90	PLK1	BIRC6
GIMAP7	ISYNA1	RPL34	UBAP1	B3GALNT2
TRD-GTC2-4	C14orf132	MECOM	ULK3	SETD7
UCHL1	DVL2	FGFR3	SNORD19	TRD-GTC2-10
MAZ	EFCAB3	SHF	C4orf29	TAGLN
FGA	CDCA8	CNNM4	SNORD6	COL6A1
MAPK1	TMCO3	SCARNA4	CDC37	DNAH17
RNA5S8	ITGB1BP1	RAB14	CDCA2	GCN1L1
FMN1	CAV1	NSUN2	TBC1D8B	CDH11
B3GALT6	RNA5S12	RNASEH1	RPL4	PSMA3
TMSB10	UTP20	CDK5RAP2	RPL3P4	UTRN
ALPK1	MCL1	CDKN3	HNRNPA0	NDC80
CHD1L	C1orf159	VPS16	SNORA29	NARF
ASAP1	BBX	VCPIP1	HDAC1	ZNF207
SNX1	SNORA63	DNAH1	SNORD114-25	TCOF1
RNA5S13	RAP1B	SNORA36B	FTH1	SRRM2
TRP-TGG1-1	RNU12	GHITM	ANKRD37	PHB2
MAPKAPK5	PPARG	NOMO1	MAPK1IP1L	IP6K1
ARL6IP5	PIGC	ATP6V1G1	ATG13	RPS25
SLC13A3	SNORD114-10	TRH-GTG1-2	HSP90AB3P	HIST1H3J
CISD2	HSP90AA1	ARID1A	RPS24	CLN8
AP2A2	DNTTIP1	FAM19A2	URGCP	MAPK10
SLC6A8	WDTC1	RPLP0P2	ELL2	DLG5
CPNE4	TXNL4A	CCNA2	ZMYND8	ATP5E
ZCCHC24	VPS37B	LINC00657	CKB	FKBP8
DVL1	PRKCA	KRT18	DUSP4	FZD2
SPRED2	LOXL2	PLXNA2	SRPK1	FDX1
AFP	KIF21B	GFPT2	TRD-GTC2-1	TCEB1
SBNO1	MED13L	MYO1C	SNORD114-24	PPAN
WHSC1	SNORD126	TRX-CAT1-7	TMX2-	ARHGAP21
RPS16	NCS1	MAPRE1	CTNND1	UBE2G1
TIMM44	EIF3G	RPS10	SNORD113-8	EIF5B
TM4SF1	COX4I1	GLTP	XRN1	RNU6-69P
RN7SL1	FAM117A	GAPDH	UBE2H	LINC00641

ITGB1	SNORA80E	YWHAG	ARPC2	LOX
LRRC8A	INPP4B	VPS26A	ANKRD50	KIF1A
GTF2H1	SNORD20	DNAJC13	DCTN5	KCNG1
HNRNPH2	SEPT7	RAD21	ITFG1	S100A10
C3	PREPL	SPECC1	ATP6V0B	VTRNA1-2
SEZ6L2	TRG-CCC2-2	PCGF1	SNORD114-22	CMTM6
SRSF3	NDUFAB1	FAM84B	RNU5B-1	WEE1
DCAF12	RBM47	RNVU1-18	TALDO1	GDE1
ANXA2	MAP1A	DYNLRB1	IQSEC3	DEK
DTX3L	BOLA2B	IER2	EHHADH	DNAJC11
BARD1	AKR1C1	SERPINA1	FNBP1	PILRB
E2F2	SLC2A3	MRPL2	VTRNA2-1	RNU5D-1
RBM8A	COL1A1	PABPC3	RPL17	ERH
CNP	FBXO24	CES2	HIST1H2BC	OAS3
AKAP2	FGR	CCDC125	HIST1H3G	HNRNPA3
RNU6-26P	CTSD	MTHFD2	SPRED1	GREB1
SNORA58	ELFN2	YY1	TRAF6	TPD52L2
SBF1	EPRS	RNY4P2	PARVA	SALL1
BPTF	ALG13	SNORD104	NPHP3-	HIST1H2BF
ABCA6	TSSK6	SERF2	ACAD11	FAM21C
SLC40A1	SIRT2	KLC1	PHIP	TOMM20
ODC1	E2F6	DENND3	SRSF1	ATAD2
UPF1	RASA1	ZCCHC4	STX6	MFF
PGLYRP3	CCDC38	RPL6	SNORD15B	TULP4
KDM1A	DPM2	PCDH7	TCAM1P	C10orf2
ATF7IP2	SRSF2	SNHG1	TRD-GTC2-5	TMEM126B
RASGEF1C	PAFAH1B1	TRAK1	TUBA1C	DSP
SNORD113-6	PLEKHM3	PTCD3	S100A11	RNF111
PLA2G16	TUBGCP2	SEC16A	SSBP1	IFT52
PNRC1	EIF3A	LRRC61	NET1	ATP6V1E1
NEK7	NECAB3	ZNF592	HIST1H2BG	KPNA4
RNA5S16	TRX-CAT1-6	RNY4P20	SCARNA3	ZFP91
CREB3L2	PCGF2	ARFGEF2	NLGN4Y	SCARNA5
NUDT21	TPI1P1	FTL	LMTK2	RNU6-30P
POLD2	MOXD2P	PRRC1	KPNB1	RPLP1
PXMP4	NMNAT3	ADCY2	LAMB2	PPIF
RNU4-1	CGGBP1	MARS2	GNG12	SNORD71
SNORD42B	TARS	COPZ1	NAA30	NOD2
RFC1	HERC1	SYNE2	TF	SPRY4
ACAP3	IFFO2	KLHL32	COPA	MEX3C
STX16	PHYHIP	MST1P2	TSPAN4	RPS6KA3
SNORD94	CSNK1A1L	MYO7A	CAPG	SLCO4C1
MST1	C16orf59	RNU7-1	SNORD111B	MAD2L2
MUC4	AHCY	HBD	SF3B1	UBE2J2

RNVU1-7
OTUD3
B4GALT5
EPB41L1
CBL
ARMCX3
SERPINF1
ADAMTSL2
MAP3K11
A2M
LINC00324
ZNF559-
ZNF177
MIF
SLC7A1
SREBF1
SNORD113-5
TRX-CAT1-8
GNA13
CPLX2
SNORD33
GNB2L1
ZFAND5
RPSA
RPL11
LMNB2
RPL23
RPS3
ZNF778
DUOXA1
SSFA2
SNORD30
SHPK
TSSK4
PTPRD
UFD1L
VRK1
TRE-TTC1-1
DAB1
HNRNPF
NPTXR
SF1
TAOK1
LYSMD4

VSNL1
PHF2P1
GFOD2
ATP2C1
LIX1L
APOO
C3orf38
CARS
SNORD41
TRG-CCC2-1
MRPS24
RPN2
LLGL2
TFPI
HBB
PITPNM3

Supplementary Table S2: Parameters for the two confounders in simulations

Samples	sEV fraction	Debris fraction	Mean of total UMI
Simulation1	10%	90%	130
Simulation2	10%	90%	100
Simulation3	10%	90%	70
Simulation4	10%	90%	40
Simulation5	5%	95%	130
Simulation6	5%	95%	100
Simulation7	5%	95%	70
Simulation8	5%	95%	40
Simulation9	1%	99%	130
Simulation10	1%	99%	100
Simulation11	1%	99%	70
Simulation12	1%	99%	40
Simulation13	0.5%	99.5%	130
Simulation14	0.5%	99.5%	100
Simulation15	0.5%	99.5%	70
Simulation16	0.5%	99.5%	40

Supplementary Table S3: Overall statistic for each scRNA-seq dataset

Study	# Samples	# Cells droplets	Mean ESAI%
Normal tissues	15	88k	1.1%
Colorectal cancer	27	40.5k	12.9%
Pancreatic ductal adenocarcinoma	27	136k	11.2%
Prostate cancer	6	20.0k	9.8%
Gastric cancer.	17	24.9k	6.0%

Supplementary Table S4: Sequencing information in each scRNA-seq sample

Samples	Study	Total reads	Saturation
SRR13075718	Normal tissues	396,889,324	0.90
SRR13075719	Normal tissues	412,330,131	0.97
SRR13075720	Normal tissues	406,559,469	0.91
SRR13075721	Normal tissues	409,911,233	0.83
SRR13075722	Normal tissues	417,665,329	0.87
SRR13075723	Normal tissues	381,569,696	0.95
SRR13075724	Normal tissues	405,595,587	0.92
SRR13075725	Normal tissues	395,499,595	0.95
SRR13075726	Normal tissues	405,745,349	0.91
SRR13075727	Normal tissues	407,153,602	0.87
SRR13075728	Normal tissues	391,514,394	0.82
SRR13075729	Normal tissues	408,635,408	0.89
SRR13075730	Normal tissues	411,719,500	0.93
SRR13075731	Normal tissues	405,264,232	0.89
SRR13075732	Normal tissues	396,476,803	0.82
scrEXT001	CRC	267,375,232	0.75
scrEXT002	CRC	275,655,234	0.73
scrEXT003	CRC	161,020,595	0.68
scrEXT009	CRC	266,870,550	0.77
scrEXT010	CRC	255,400,094	0.80
scrEXT011	CRC	149,987,327	0.68
scrEXT012	CRC	173,029,484	0.69
scrEXT013	CRC	148,821,159	0.73
scrEXT014	CRC	126,457,430	0.78
scrEXT015	CRC	202,089,559	0.82
scrEXT016	CRC	125,310,265	0.53
scrEXT017	CRC	313,368,372	0.78
scrEXT018	CRC	200,491,544	0.55
scrEXT019	CRC	158,677,458	0.57
scrEXT020	CRC	163,981,182	0.65
scrEXT021	CRC	73,852,856	0.84
scrEXT022	CRC	69,557,278	0.65
scrEXT023	CRC	102,694,399	0.77
scrEXT024	CRC	60,655,914	0.70
scrEXT025	CRC	135,684,628	0.63
scrEXT026	CRC	119,764,778	0.66
scrEXT027	CRC	111,193,987	0.57
scrEXT028	CRC	128,496,926	0.59
scrEXT029	CRC	179,638,932	0.68
scrEXT030	CRC	113,336,320	0.97
scrEXT031	CRC	223,194,973	0.83
scrEXT032	CRC	105,182,173	0.76

SRX6887739	PC	344,550,740	0.54
SRX6887740	PC	377,754,404	0.79
SRX6887741	PC	409,843,799	0.84
SRX6887742	PC	396,675,758	0.84
SRX8890105	PC	390,209,140	0.60
SRX8890106	PC	467,109,851	0.50
CRR034499	PDAC	475,639,068	0.91
CRR034500	PDAC	503,646,305	0.94
CRR034501	PDAC	456,377,876	0.88
CRR034503	PDAC	424,575,816	0.89
CRR034504	PDAC	432,664,967	0.82
CRR034505	PDAC	462,415,046	0.95
CRR034506	PDAC	422,634,242	0.85
CRR034507	PDAC	469,792,823	0.89
CRR034509	PDAC	447,716,125	0.85
CRR034510	PDAC	454,620,021	0.81
CRR034511	PDAC	437,501,629	0.86
CRR034512	PDAC	358,926,319	0.63
CRR034513	PDAC	361,887,534	0.74
CRR034516	PDAC	476,052,968	0.92
CRR034517	PDAC	464,724,010	0.87
CRR034519	PDAC	427,255,401	0.82
CRR034520	PDAC	433,420,379	0.83
CRR034521	PDAC	456,175,874	0.85
CRR034522	PDAC	452,602,094	0.95
CRR034523	PDAC	458,593,154	0.78
CRR034524	PDAC	434,672,976	0.91
CRR034525	PDAC	426,347,908	0.80
CRR034526	PDAC	435,434,526	0.88
CRR034527	PDAC	444,737,664	0.83
CRR034528	PDAC	445,270,923	0.79
CRR034529	PDAC	127,067,667	0.72
CRR034530	PDAC	461,733,524	0.86
5846n1	GC	63,946,877	0.71
5846t1	GC	66,269,940	0.60
5866n1	GC	178,796,565	0.79
5866n2	GC	186,221,052	0.76
5866t1	GC	85,461,675	0.53
5866t2	GC	215,238,480	0.82
5931PBMC	GC	243,060,396	0.90
6207n1	GC	128,921,802	0.75
6207PBMC	GC	214,357,635	0.85
6207t1	GC	98,643,150	0.66
6342n1	GC	30,976,590	0.72

6592n1	GC	63,309,496	0.74
6592t1	GC	550,807,424	0.88
6649t1	GC	42,429,204	0.91
6649n1	GC	55,789,778	0.92
6709n1	GC	229,024,484	0.95
6709t1	GC	99,845,312	0.92

CRC: colorectal cancer; PDAC: pancreatic ductal adenocarcinoma; PC: prostate cancer; GC: gastric cancer.
