# Supplementary material for: SEVtras delineates small extracellular vesicles at droplet resolution from single-cell transcriptomes
Source: Nat Methods. 2023 Dec 4;21(2):259–66. doi: 10.1038/s41592-023-02117-1 (PMC10864178; doi:10.1038/s41592-023-02117-1)
Supplement: Supplementary file 2 — Reporting Summary [file 41592_2023_2117_MOESM2_ESM.pdf]

nature portfolio

Corresponding author(s): Fangqing Zhao

Last updated by author(s): Sep 12, 2023

Reporting Summary

Nature Portfolio wishes to improve the reproducibility of the work that we publish. This form provides structure for consistency and transparency in reporting. For further information on Nature Portfolio policies, see our Editorial Policies and the Editorial Policy Checklist.

Statistics

For all statistical analyses, confirm that the following items are present in the figure legend, table legend, main text, or Methods section.

n/a	Confirmed	
☐	☒	The exact sample size (*n*) for each experimental group/condition, given as a discrete number and unit of measurement
☐	☒	A statement on whether measurements were taken from distinct samples or whether the same sample was measured repeatedly
☐	☒	The statistical test(s) used AND whether they are one- or two-sided<br>*Only common tests should be described solely by name; describe more complex techniques in the Methods section.*
☒	☐	A description of all covariates tested
☐	☒	A description of any assumptions or corrections, such as tests of normality and adjustment for multiple comparisons
☐	☒	A full description of the statistical parameters including central tendency (e.g. means) or other basic estimates (e.g. regression coefficient) AND variation (e.g. standard deviation) or associated estimates of uncertainty (e.g. confidence intervals)
☒	☐	For null hypothesis testing, the test statistic (e.g. *F*, *t*, *r*) with confidence intervals, effect sizes, degrees of freedom and *P* value noted<br>*Give P values as exact values whenever suitable.*
☒	☐	For Bayesian analysis, information on the choice of priors and Markov chain Monte Carlo settings
☒	☐	For hierarchical and complex designs, identification of the appropriate level for tests and full reporting of outcomes
☐	☒	Estimates of effect sizes (e.g. Cohen's *d*, Pearson's *r*), indicating how they were calculated

Our web collection on statistics for biologists contains articles on many of the points above.

Software and code

Policy information about availability of computer code

Data collection	Public data were downloaded from NCBI/ArrayExpress/NGDC/customed websites through wget (v1.12).
Data analysis	Our proposed method SEVtras is available at https://github.com/bioinfo-biols/SEVtras (v 0.3). For RNA-seq data, raw reads were cleaned using Trim Galore (v0.6.7) and aligned to the GRCh38 human reference genome using STAR (v2.6.1a) and RSEM (v1.2.25) to quantify the transcripts. For scRNA-seq data, raw reads were processed by Cell Ranger (v5.0.0) and downstream single-cell analysis was performed with Scanpy (v1.8.2), MAGIC (v3.0.0) and BBKNN (v1.5.1). All data were analyzed using python 3.8 with numpy (v1.20.3) and pandas (v1.2.4), and visualized using matplotlib (v3.4.2) and seaborn (v0.11.0).

For manuscripts utilizing custom algorithms or software that are central to the research but not yet described in published literature, software must be made available to editors and reviewers. We strongly encourage code deposition in a community repository (e.g. GitHub). See the Nature Portfolio guidelines for submitting code & software for further information.

Data

Policy information about availability of data

All manuscripts must include a data availability statement. This statement should provide the following information, where applicable:

- Accession codes, unique identifiers, or web links for publicly available datasets
- A description of any restrictions on data availability
- For clinical datasets or third party data, please ensure that the statement adheres to our policy

The bulk RNA-seq and scRNA-seq data for MSC and 293F cells were deposited at NGDC with accession number PRJCA017291 (https://ngdc.cncb.ac.cn/gsa-human/browse/HRA004708). The CITE-seq data and scRNA-seq data for normal tissues and prostate cancer were downloaded from the NCBI Gene Expression Omnibus (GSE150599, GSE159929 and GSE137829, respectively). The scRNA-seq data for colorectal cancer were accessed in ArrayExpress under accession number E-MTAB-8410. The scRNA-seq data for pancreatic ductal adenocarcinoma were accessed in GSA with the accession number CRA001160. The scRNA-seq data for gastric cancer were accessed at https://dna-discovery.stanford.edu/research/datasets/.

Human research participants

Policy information about studies involving human research participants and Sex and Gender in Research.

Reporting on sex and gender	The study did not involve sex and gender.
Population characteristics	The study did not recruit participants, as we used public datasets from previous studies.
Recruitment	The study did not recruit participants, as we used public datasets from previous studies.
Ethics oversight	The study did not involve ethics, as we used public datasets from previous studies.

Note that full information on the approval of the study protocol must also be provided in the manuscript.

Field-specific reporting

Please select the one below that is the best fit for your research. If you are not sure, read the appropriate sections before making your selection.

☒ Life sciences ☐ Behavioural & social sciences ☐ Ecological, evolutionary & environmental sciences

For a reference copy of the document with all sections, see nature.com/documents/nr-reporting-summary-flat.pdf

Life sciences study design

All studies must disclose on these points even when the disclosure is negative.

Sample size	No sample size calculations were performed. For RNA-seq, we only used cell lines for sequencing and all samples were replicated with two biological replicates to ensure reproducibility. For scRNA-seq, we first only performed on cell lines, all of which had >1,000 cells. We then collected six public large-scale scRNA-seq datasets comprising > 90 samples, which should be sufficient as in most single-cell studies.
Data exclusions	No data were excluded from the analyses.
Replication	We generated two biological replicates for all conditions explored in our study. All attempts were confirmed to be successful.
Randomization	Randomization was not relevant to our study, as we used public datasets from previous studies and no group assignment was needed.
Blinding	Blinding was not necessary for the same reason as no objective scoring was applied in our study.

Reporting for specific materials, systems and methods

We require information from authors about some types of materials, experimental systems and methods used in many studies. Here, indicate whether each material, system or method listed is relevant to your study. If you are not sure if a list item applies to your research, read the appropriate section before selecting a response.

Materials & experimental systems

n/a	Involved in the study	
☐	☒	Antibodies
☐	☒	Eukaryotic cell lines
☒	☐	Palaeontology and archaeology
☒	☐	Animals and other organisms
☒	☐	Clinical data
☒	☐	Dual use research of concern

Methods

n/a	Involved in the study	
☒	☐	ChIP-seq
☒	☐	Flow cytometry
☒	☐	MRI-based neuroimaging

Antibodies

Antibodies used	The antibodies used for western blot:<br>1. Rabbit polyclonal anti-CD9, Proteintech, Cat# 20597-1-AP.<br>2. Rabbit polyclonal anti-Syntenin-1, Proteintech, Cat# 22399-1-AP.<br>3. Rabbit polyclonal anti-Calnexin, EASYBIO, Cat# BE3386.<br>4. Rabbit polyclonal anti-GRP94, Proteintech, Cat# 14700-1-AP.<br>5. HRP-conjugated goat anti-rabbit antibody, EASYBIO, Cat# BE0101.
Validation	Validation was relied on the available data provided by the manufacture's websites for all antibodies:<br>1. Proteintech claims that this Rabbit polyclonal anti-CD9 (Cat# 20597-1-AP) is suitable for WB applications in human. (https://www.ptgcn.com/products/CD9-Antibody-20597-1-AP.htm)<br>2. Proteintech claims that this Rabbit polyclonal anti-Syntenin-1 (Cat# 22399-1-AP) is suitable for WB applications in human. (https://www.ptgcn.com/products/SDCBP-Antibody-22399-1-AP.htm)<br>3. EASYBIO claims that this Rabbit polyclonal anti-Calnexin (Cat# BE3386) is suitable for WB applications in human. (http://bioeasytech.com/product/2629.html?goods_id=4824)<br>4. Proteintech claims that this Rabbit polyclonal anti-GRP94 (Cat# 14700-1-AP) is suitable for WB applications in human. (https://www.ptgcn.com/products/HSP90B1-Antibody-14700-1-AP.htm)<br>5. EASYBIO claims that this HRP-conjugated goat anti-rabbit antibody (Cat# BE0101) is suitable for WB applications to detect rabbit primary antibody. (http://www.bioeasytech.com/product/2901.html?goods_id=5786)

Eukaryotic cell lines

Policy information about cell lines and Sex and Gender in Research

Cell line source(s)	MSC cells were provided by Jinan Wanquan Biotechnology (Shandong, China). 293F cells were originally acquired from QuaCell Biotechnology (Guangdong, China).
Authentication	MSC and 293F cell lines were authenticated by STR DNA profiling analysis.
Mycoplasma contamination	MSC and 293F cell lines were tested negative for mycoplasma contamination.
Commonly misidentified lines<br>(See ICLAC register)	No commonly misidentified cell line was used in this study.

March 2021
