# Supplementary figures and images for: SEVtras delineates small extracellular vesicles at droplet resolution from single-cell transcriptomes
Source: Nat Methods. 2023 Dec 4;21(2):259–66. doi: 10.1038/s41592-023-02117-1 (PMC10864178; doi:10.1038/s41592-023-02117-1)

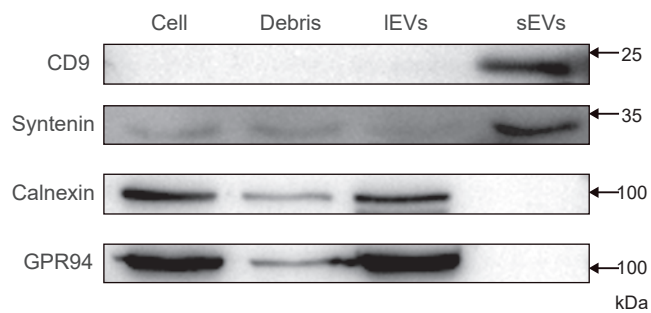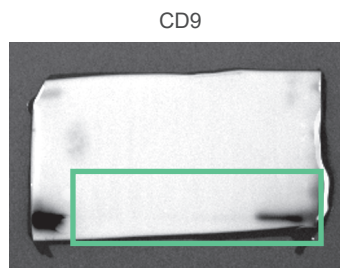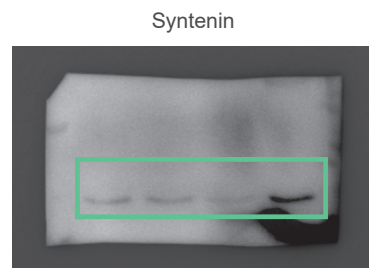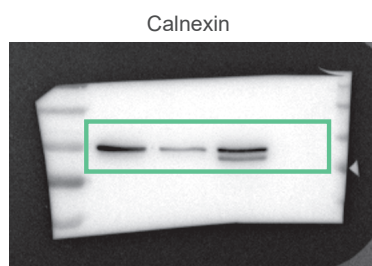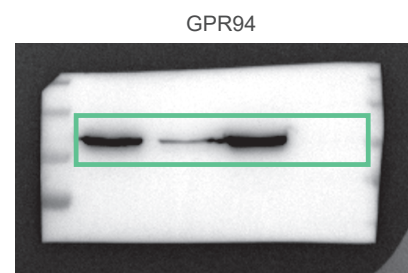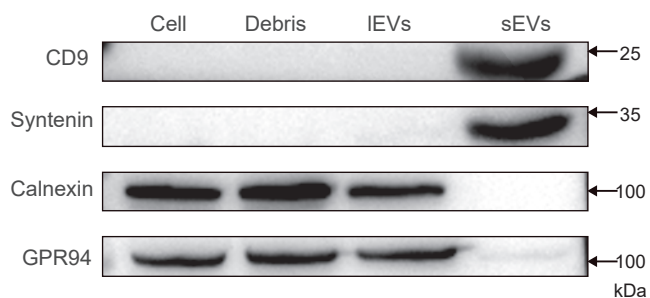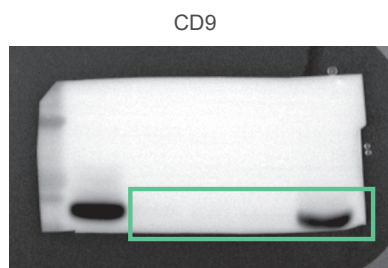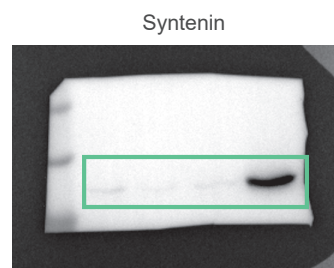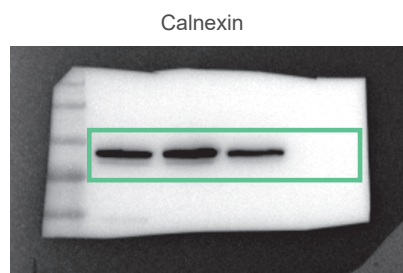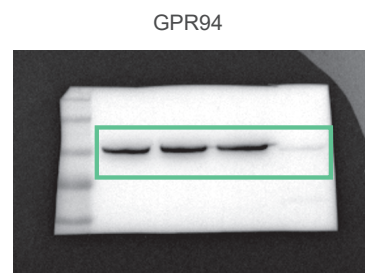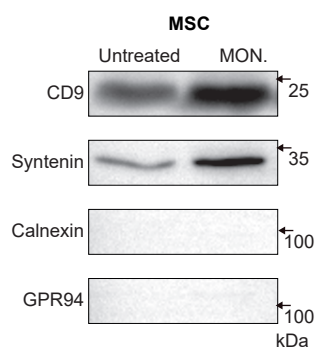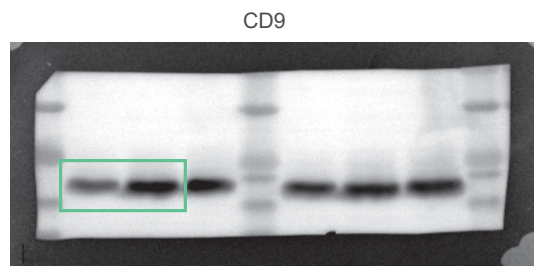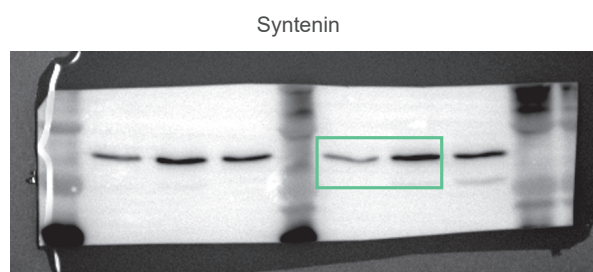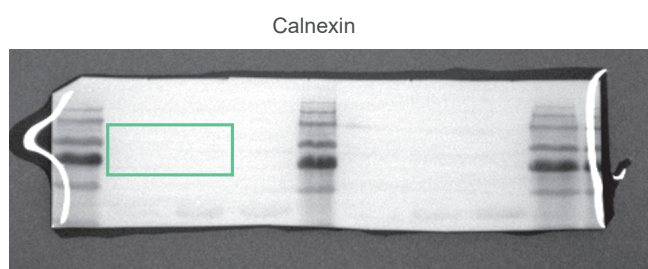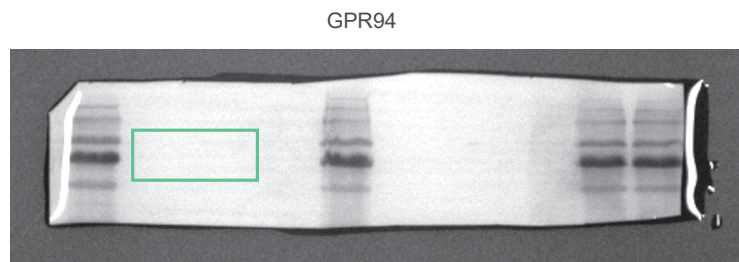

Supplement: Supplementary file 4 — Unprocessed western blots. [file 41592_2023_2117_MOESM4_ESM.pdf]
